# To Fully Tackle the Gang of Four, Needs-Driven R & D Is Essential

**DOI:** 10.1371/journal.pmed.0030282

**Published:** 2006-06-27

**Authors:** Els Torreele, Catherine Royce, Robert Don, Ann-Marie Sevcsik, Simon Croft

**Affiliations:** **1**Drugs for Neglected Diseases Initiative, GenevaSwitzerland

We congratulate Hotez et al. [ 1] for a compelling and important paper that challenges the global health audience to address neglected tropical diseases affecting the poor and powerless in resource-poor settings. Full support should be given to the call for a global strategy to tackle the neglected “gang of four,” which will add the most neglected tropical diseases to the well-known big three (HIV/AIDS, malaria, and TB).

For those neglected diseases for which adequate tools exist, a strategy of integrated chemotherapy linked to the big three could be the best way to reduce disease burden and disease-related deaths, as is convincingly argued by the authors in the case of helminth infections. However, this is a simplification of the situation we face for other neglected tropical diseases such as human African trypanosomiasis (HAT), Chagas disease, and Buruli ulcer. No adequate tools exist to diagnose and treat these fatal or severely debilitating conditions. The top priority therefore should be new and innovative research and development (R & D) to develop adequate treatments: simple and cheap diagnostics and safe, efficacious, easy-to-use, and affordable medicines.

In the case of HAT, current diagnosis is cumbersome and invasive, while the few existing drugs are old, toxic, difficult to use, and increasingly ineffective [ 2]. For visceral leishmaniasis, another fatal disease when left untreated, major treatment complications are invasive diagnostics, long durations of treatment (30 d), and drug resistance (up to 40% in India) [ 3]. No drugs even exist to treat patients with Buruli ulcer or chronic Chagas disease.

Several new initiatives are beginning to address these challenges. For instance, the Drugs for Neglected Diseases initiative (DNDi) ( http://www.dndi.org) was launched in 2003 to develop new and field-adapted treatments for neglected diseases like HAT, leishmaniasis, and Chagas disease. The Foundation for Innovative New Diagnostics recently initiated a new HAT diagnostics programme, and several groups in academia and pharma are increasing their R & D efforts in neglected tropical diseases. However, this is just a start. These efforts will only come to fruition if sustained support can be mobilized.

Needs-driven R & D for new tools is essential, as is government support for both R & D and implementation of effective interventions when available. In 2005, DNDi launched an international R & D appeal ( http://www.researchappeal.org) that urges governments to set global public health priorities, to fund R & D for neglected diseases, and to provide new rules to stimulate essential health R & D. This effort builds on momentum gained over the past years to provide an international response to correct the fatal imbalance of adequate health tools for neglected diseases.

We hope all governments will sustain this momentum in May 2006 at the World Health Assembly, which will consider an essential health R & D resolution calling for a global framework to support needs-driven research and to set R & D priorities in the interest of public health, especially for the most neglected diseases [ 4]. The G8 summit in July provides an opportunity for those governments to financially commit to their 2005 pledge to support drug R & D for neglected diseases.

Hotez and colleagues have offered original proposals to increase the effectiveness of existing tools in the control of certain neglected tropical diseases outside of the big three. But to really tackle the gang of four, adequate and field-adapted health tools must be available, and governments must prioritize needs-driven R & D for those diseases where no such tools exist.
